# Identified Motivation as a Key Factor for School Engagement During the COVID-19 Pandemic-Related School Closure

**DOI:** 10.3389/fpsyg.2021.752650

**Published:** 2021-11-16

**Authors:** Léa Tân Combette, Etienne Camenen, Jean-Yves Rotge, Liane Schmidt

**Affiliations:** ^1^Control-Interoception-Attention Team, Institut du Cerveau (ICM), INSERM UMR 1127, CNRS UMR 7225, Sorbonne Université, Paris, France; ^2^iCONICS Team, Institut du Cerveau (ICM), INSERM UMR 1127, CNRS UMR 7225, Sorbonne Université, Paris, France; ^3^Service de Psychiatrie d'Adultes, APHP Sorbonne Université, site Pitié-Salpêtrière, Paris, France

**Keywords:** motivation, school engagement, COVID-19, middle school, self-regulated

## Abstract

On March 16, 2020, French schools suddenly closed due to the COVID-19 pandemic, and middle school students were asked to study from home with no direct interactions with teachers or classmates. However, school plays an important role in the development of social, intellectual, and mental competencies and can counteract the negative effects of adverse life events on learning and early school dropout. In this study, we investigated how the unusual context of school closure during the COVID-19 pandemic affected school engagement. Specifically, we focused on inter-individual differences in the motivational determinants of school engagement. We thus performed an online survey of 170 students focusing on the time spent on mathematics assignments, motivation regulation, implicit theories of intelligence, such as adopting a growth or a fixed mindset about his/her intellectual abilities, and optimism. Importantly, the students participated in the online survey during the first lockdown period, with schools closed (T1), and the second lockdown period, with schools remaining open (T2). During T1, identified motivation positively predicted the time spent on math homework assignments: the more the students thought that working on math exercises was useful for their future life, the more time they spent studying. Importantly, the link between identified motivation and school engagement was specific to T1, when schools were closed, as indicated by a significant interaction between identified motivations by type of lockdown. These results suggest that having self-determined motivation is of particular importance when students are deprived of social and intellectual interactions with classmates and teachers. This finding paves the way toward the development of wise rational interventions that target identified motivation and can be applied during challenging societal times and adverse, common life events to keep students engaged with school.

## Introduction

On January 30, 2020, the WHO declared the spread of the acute respiratory syndrome-causing severe acute respiratory syndrome coronavirus 2 (SARS-CoV-2) across the world to be a public health emergency and warned of a global pandemic (World Health Organization, 2020). In the following weeks, strict lockdowns, with school closures, were implemented in many countries throughout the world to contain the spread of the virus. However, it also left many households unprepared for dealing with the situation. For example, in France, the experience of the COVID-19 pandemic-related school closures differed between households, depending on access to the Internet and computer equipment (Institut National de la Statistique et des Etudes Economiques, 2020). Studies from education and social psychology have shown that experiencing long periods of school closure has negative effects on learning, motivation, and psychosocial well-being of the students (Sprang and Silman, 2013; Benke et al., 2020a,b; Dorn et al., 2020; Guessoum et al., 2020; Magklara et al., 2020; UNESCO, 2020; Bernabe-Valero et al., 2021; Garcia-Esquinas et al., 2021; Rajmil et al., 2021). Their direct interaction with friends and teachers at school has also been shown to counteract the negative effects of stressful life events on the well-being and mental health of the students (Shahar et al., 2009). This is quite important, as stressful life events are closely linked to early school dropout and demotivation (Dupéré et al., 2018). It is therefore critical to identify the individual factors that contribute to school engagement when schools are closed during adverse life events. This is important because it would make it possible to detect those students who are at risk of the negative consequences of pandemic-related lockdowns on health or school dropout and to design interventions that could help in keeping such students engaged. Although it is still unknown how inter-individual differences may contribute to school engagement during the pandemic-related lockdown, we tested three main individual characteristics that are associated with school engagement, namely, motivation regulation, intelligence mindset, and optimism.

First, an important body of research has shown that different types of motivational states or regulations can influence school engagement. Notably, the self-determination theory of motivation proposes that behavior is regulated by goals that can be placed on a spectrum from intrinsic to extrinsic (Deci and Ryan, 1985, 2012). For example, motivation of a student to do homework can be driven by external regulators, such as obtaining good grades or meeting the demands of parents. It can also be driven by identified regulators, such as the desire to act in coherence with his/her own attitudes, values, and needs, or by intrinsic regulators, such as curiosity and interest. The intrinsic and identified regulations (IR) of motivation have been shown to positively affect school achievement and have a more sustained effect on school engagement, whereas extrinsic regulators are associated with more maladaptive outcomes (Vasconcellos et al., 2020; Guay et al., 2021). However, intrinsic and identified motivation can also have different effects. Burton et al. (2006) showed that IR is positively associated with both greater well-being and better grades. In contrast, intrinsically motivated students have been shown to experience greater well-being after their mid-term exams, regardless of the grades obtained. Consistent with these results, Liu et al. (2019) provided longitudinal evidence that intrinsic motivation (IM) positively affects school performance in the long term, whereas identified motivation has a more acute, short-term performance-enhancing effect.

Second, another stream of research has shown that school engagement is strongly determined by the implicit theories of intelligence (ITIs) (Dweck, 1986, 2008; Dweck and Leggett, 1988). ITI proposes that humans hold strong beliefs about how malleable their intelligence is from very early on. Such beliefs are also referred to as fixed or growth mindsets. Students who adopt a fixed mindset of intelligence believe that intelligence is inherent and does not change during their lifetime. On the contrary, students who adopt a growth mindset of intelligence believe that intelligence is malleable and can evolve throughout life with experience and training. Importantly, these mindsets have an impact on the attitudes of students toward school. Students with a growth mindset are more prone to adopt learning goals that are defined by the intention to learn new things, rather than performance goals (Dweck, 1986; Blackwell et al., 2007). Adopting a growth mindset over a fixed mindset has also been found to positively affect school performance and well-being (Claro et al., 2016; Sarrasin et al., 2018; Sethi and Shashwati, 2019). Furthermore, mindset is particularly important for performance in mathematics because a fixed mindset concerning math skills has been shown to be more frequent among students and teachers than for other disciplines (Gunderson et al., 2017).

Third, it has been shown that optimism protects against school dropout through its effect on motivation and adjustment to stressors (Hoy et al., 2006; Huan et al., 2006; Solberg Nes et al., 2009; Tetzner and Becker, 2018). More precisely, optimistic students are more motivated to persist because they have better expectations concerning outcomes and cope better with stressors by managing, decreasing, or eliminating them. In mathematics, optimism has been associated with achievement, as pessimistic students have already experienced lower achievement in primary school (Yates, 2002). In older students, optimism predicts better adjustment of students to the transition from high school to college (Chemers et al., 2001).

We explored how (1) motivation regulation, (2) intelligence mindset, and (3) optimism impacted the personal time students engaged in mathematical exercises with the aim to assess how inter-individual differences may have contributed to school engagement during the pandemic-related lockdown. We focused on time spent on mathematics homework assignments because helping middle school students to achieve a good level in mathematics is a daily and complex mission, especially when schools are closed. The 2019 Program for International Student Assessment (PISA) study has shown that the proficiency of French middle school students in mathematics is among the lowest of European countries (Schleicher, 2019). Numeracy skills are a source for inter-individual differences in income, health, and unlawful behavior (Parsons and Bynner, 2005; Butterworth et al., 2011). We thus conducted an online study across students who attended the 6th−9th grades of middle school in the Paris area. The data were collected in two groups at different periods during the COVID-19 pandemic in 2020. This year was characterized by two major waves of SARS-CoV-2 contagion in France, leading to strict lockdowns of economic and social life, with major individual restrictions in everyday life. However, the two lockdowns differed by one factor, i.e., school closure. The first lockdown period from March to June 2020 involved school closures and homeschooling, whereas schools remained open during the second lockdown period from October to December 2020. This provided us with the unique opportunity to investigate the effects of closed schools on school engagement by keeping other confounding contextual factors of the lockdown constant.

## Materials and Methods

### Ethical Considerations

The study protocol was approved by the Academy of Versailles and was conducted in accordance with the Declaration of Helsinki. All participants and their parents gave their informed consent.

### Participants

Participants were recruited from eight different public middle schools in Paris, selected, and governed by the Academy of Versailles. The principals of the eight selected middle schools agreed to send the questionnaire to their students by email. Participants all attended the 6th−9th grades. In total, 276 middle school students participated in an online survey study. Among them, 183 (mean age ± SEM = 12.9 ± 0.1 years, range = 10–15 years old) provided complete survey responses. From these sample data, 13 students were excluded because the individual survey responses were identified as being outliers, scoring three SDs above or below the sample mean of the respective variables. Therefore, a total sample size of 170 responses was included, with 97 responses for group 1, who were tested during the first lockdown period (T1, April to June 2020), with schools closed, and 73 responses for group 2, who were tested during the second lockdown period (T2, November to December 2020), with schools opened (Table 1). During the lockdown with schools closed, middle schools were recruited at the end of the lockdown period, allowing us to collect data during the last month of school closure. During the lockdown with schools opened, middle schools were recruited earlier, allowing us to collect data during the entire lockdown period. Among the participants, 30 participants were from 6th grade, 49 from 7th grade, 38 from 8th grade, and 53 from 9th grade.

**Table 1:** Lockdown conditions were comparable in T1 and T2, except that schools were closed during the first lockdown and open during the second one.

**Restrictions**	**1st Lockdown (T1)** **April to June 2020**	**2d Lockdown (T2)** **November to December 2020**
Middle Schools	Closed	Open
Work life	Remote	Remote
Shops and restaurants	Closed, except for alimentary shops	Closed, except for alimentary shops
Cultural and recreational places	Closed	Closed

Participants came from eight different middle schools. All analyses were, therefore, controlled for potential differences in time spent on math homework between the schools by entering the schools as a random effect in all linear mixed-effect analyses. Moreover, the two testing period groups were matched for general success rates in the national, final middle school examinations (e.g., “Brevet des Collèges”), as indicated by a non-significant difference in success rates [*t*_(6)_ = 0.65, *p* = 0.27, two-sampled, one-tailed *t*-test]. This success rate refers to the percentage of students who attained the minimum score necessary to pass the national examination at the end of middle school. This percentage of students can be consulted online and can act as a ranking of middle schools on quality and school performance.

Moreover, the two groups (T1 and T2) were matched in terms of average age [*t*_(168)_ = 0.43, *p* = 0.67 two-sampled, one-tailed *t*-test], level of IM [*t*_(168)_ = −0.49, *p* = 0.69], IR [*t*_(168)_ = 0.49, *p* = 0.69], extrinsic regulation [ER, *t*_(168)_ = −1.03, *p* = 0.85], growth mindset [*t*_(168)_ = 0.59, *p* = 0.72], and optimism [*t*_(168)_ = 0.22, *p* = 0.59].

### Online Survey

The study was designed as an online survey using Qualtrics software (Qualtrics, Provo, UT, USA). The survey link was sent to the participants *via* an intranet message (PRONOTE, Index Education) by the principal of each middle school, respectively. Students responded using their home computer, smartphone, or tablet.

The survey involved 33 questions measuring the following variables:

#### School Engagement in Mathematics

To measure the mathematics engagement, students rated how much time they spent doing math homework assignments each day during the previous 7-day week. They used a 5-point Likert scale from 0 (no work in Math) to 4+ (4 h of work or more) for each day of the previous week.

The time spent by students on mathematics was averaged across all days (mean total ± SEM = 3.6 ± 2.6 h/week, range = 0–14 h/week; Table 2 for group differences) and square-root transformed to improve the normality of residuals in the statistical analyses.

**Table 2:** Linear mixed effects model of time spent on math homework in *N* = 170 middle school students tested during two periods of COVID-19 related lockdown with schools closed (group 1) and open (group 2).

**Fixed effects**	**β_i_**	**SE**	**t**	***p***	**95%CI**
(Intercept)	1.74	0.09	20.01	4.4e-03	1.59–1.91
Group	−0.03	0.07	−0.49	0.63	−0.15–0.09
Intrinsic	0.09	0.07	1.27	0.21	−0.04–0.20
Extrinsic	−0.01	0.06	−0.21	0.83	−0.12–0.10
**Identified**	**0.18**	**0.08**	**2.31**	**0.02**	**0.04–0.32**
Mindset	2.1e-03	0.06	0.03	0.97	−0.11–0.11
Optimism	−0.11	0.06	−1.79	0.07	−0.23–0.00
**Grade**	**−0.08**	**0.04**	**−2.04**	**0.04**	**−0.16–0.01**
Group by Intrinsic	−0.02	0.06	−0.29	0.77	−0.13–0.09
Group by Extrinsic	0.08	0.06	1.32	0.19	−0.02–0.20
**Group by Identified**	**−0.14**	**0.07**	**−2.02**	**0.04**	**−0.27–0.02**
Group by Mindset	−0.09	0.06	−1.57	0.12	–0.2–0.02
Group by Optimism	0.05	0.06	0.88	0.38	−0.05–0.17
Group by Grade	0.06	0.04	1.46	0.15	−0.02–0.13
Intrinsic by Extrinsic	0.02	0.08	0.28	0.78	−0.12–0.16
Intrinsic by Identified	0.02	0.07	0.37	0.71	−0.10–0.15
Intrinsic by Mindset	5.8e-03	0.07	−0.08	0.93	−0.13–0.13
Intrinsic by Optimism	−0.11	0.06	−1.77	0.08	−0.22–0.00
Intrinsic by Grade	−1.8e-03	0.04	−0.04	0.96	−0.08–0.08
Extrinsic by Identified	0.10	0.07	1.36	0.18	−0.03–0.24
Extrinsic by Mindset	0.06	0.06	0.97	0.33	−0.06–0.16
Extrinsic by Optimism	−0.11	0.06	−1.62	0.11	−0.22–0.02
Extrinsic by Grade	0.02	0.04	0.53	0.59	−0.05–0.10
Identified by Mindset	−0.04	0.08	−0.51	0.61	−0.17–0.11
Identified by Optimism	0.11	0.06	1.64	0.10	−0.01–0.22
Identified by Grade	0.03	0.04	0.69	0.49	−0.05–0.11
Mindset by Optimism	0.07	0.06	1.20	0.23	−0.04–0.19
Mindset by Grade	−8.7e-03	0.04	−0.20	0.84	−0.08–0.07
Optimism by Grade	0.05	0.04	1.11	0.27	−0.03–0.13
**Random Effects**					
		Variance			
School (8 levels)	(Intercept)	2.3e-09			
Days Off (4 levels)	(Intercept)	1.0e-02			
Residuals	0.53	0.53			
Observations = 170; REML = 460.8					

*Group designates a regressor that compared students tested during the first COVID-19 related lockdown period with schools closed (group 1) to students tested during a second COVID-19 related lockdown period with schools open (group 2). Significant main effects and interactions are highlighted in bold*.

#### Motivation Regulation

To assess how motivation in doing math homework assignments was regulated, students completed the French version of the Elementary School Motivation Scale (Guay et al., 2010). The questionnaire involved nine items measuring IM, identified (IR), and extrinsic regulation (ER). For each item, participants had to rate whether they agreed to the statements on a binary “Yes/No” scale (e.g., “In life, it is important to learn how to do math” or “I do math to please my parents or my teacher”). “Yes” responses for each statement were summed to yield the total scores, ranging from 0 to 3, for intrinsic (*N* = 170, mean ± SEM = 1.6 ± 0.09), identified (mean ± SEM = 2.6 ± 0.05), and extrinsic motivation (mean ± SEM = 1.03 ± 0.07). All scores were *z*-scored for the analyses. Notably, we explored different motivational profiles within the sample and how they related to school engagement when schools were closed and when they were opened by conducting a cluster analysis, which is reported in the Supplementary Material.

#### Implicit Theories of Intelligence (ITIs)

The ITIs were assessed using the 3-Item Growth Mindset Scale (Dweck, 1999, 2006). Participants had to indicate to what degree they agreed with the three statements using a 6-point Likert scale, ranging from 1 (strongly agree) to 6 (strongly disagree).

Notably, we replaced “tu” (“you”) with “je” (“I”) in the French version to ensure that responses of students reflected how much they think their intelligence can grow with training, rather than merely reciting scientific facts about intelligence (De Castella and Byrne, 2015).

Scores for ITIs were obtained by averaging the scores for the three items. Higher scores indicate stronger growth mindsets (*N* = 170, mean ± SEM = 3.8 ± 0.1, range = 1–6). The scores were *z*-scored for the analyses.

#### Optimism

Optimism was measured using the Revised Life Orientation Test Scale (LOT-R; Scheier et al., 1994), which is frequently used to assess optimism and pessimism in adults and adolescents (Creed et al., 2002). The questionnaire involved 10 statements about the future (Supplementary Material), and participants rated how much they agreed with these statements using a 5-point Likert scale ranging from 0 (strongly disagree) to 4 (strongly agree). Scores were obtained by summing the responses of six non-filler items (*N* = 170, mean ± SEM = 13.88 ± 0.28, range = 0–24), with higher scores indicating more optimistic views of the future and lower scores indicating more pessimistic views. The scores were *z*-scored for the analyses.

#### Demographic Variables

At the end of the survey, students indicated their age, grade, and school and could declare if they did not understand some of the questionnaire items. Following the recommendations of the French National Commission for Information Technology and Individual Freedom (CNIL, 2013), we did not collect information about gender because crossing information about the middle schools, grade, and age with gender could threaten the anonymization of the data.

### Statistical Analyses

All statistical tests were conducted using R (RStudio Team, 2019). Three analyses were performed. The first analysis directly compared the two samples to test our main question, i.e., how school closure during the COVID-19-related lockdown influenced school engagement in mathematics and interacted with psychological variables such as type of motivation, optimism, and growth mindsets. Then, a second and a third analysis, respectively, were performed to describe each sample more precisely. Our approach consisted first of a group comparison to test how school closure influenced (1) how much the students engaged in math assignments and (2) to what extent this engagement was predicted by intrinsic, extrinsic, or identified regulators of motivation, mindset, and optimism. We thus performed a linear mixed-effect regression analysis using the fitlme function of the lmerTest package in R. As shown in equation 1 below, the model fitted the time spent on mathematics (SqMATH, square-root transformed) and included the following fixed effects for Group (i.e., group 1, which was tested during the lockdown period with schools closed, coded −1 and group 2, which was tested during the lockdown period with schools open, coded 1), IR (*z*-score), IM (*z*-score), growth mindset (*z*-score), optimism (*z*-score), and grade (coded −2, −1, 1, and 2 for the 6th−9th grades). Importantly, the comparisons of interest involved fixed effects regressors for the interaction between group and regulation type (identified, intrinsic, and extrinsic), mindset, and optimism, respectively (highlighted in bold, equation 1). The model further controlled for fixed effects of the interactions between group and grade, between different types of regulation (intrinsic, identified, or extrinsic), between different types of regulation and optimism, mindset, or grade, between the level of optimism and mindset or grade, and between mindset and grade. Two random effects regressors nested the intercept by the number of holidays and the middle school to control for these two potential confounders across all participants.


(1) $$\begin{array}{l} \text{SqMATH}=\text{Group}+\text{Intrinsic}+\text{Identified}+\text{Extrinsic}\\ +\text{Mindset}+\text{Optimism}+\text{Grade}+\text{Group }*\text{ Intrinsic}\\ +\text{Group }*\text{ Identified}+\text{Group }*\text{ Extrinsic}+\text{Group }*\text{ Optimism}\\ +\text{Group }*\text{ Mindset}+\text{Group }*\text{ Grade}+\text{Intrinsic }*\text{ Identified}\\ +\text{Intrinsic }*\text{ Extrinsic}+\text{Identified }*\text{ Extrinsic}\\ +\text{Intrinsic }*\text{ Mindset}+\text{Intrinsic }*\text{ Optimism}\\ +\text{Intrinsic }*\text{ Grade}+\text{Intrinsic }*\text{ Group}+\text{Identified }*\text{ Mindset}\\ +\text{Identified }*\text{ Optimism}+\text{Identified }*\text{ Grade}\\ +\text{Identified }*\text{ Group}+\text{Extrinsic }*\text{ Mindset}+\text{Extrinsic }*\text{ Optimism}\\ +\text{Extrinsic }*\text{ Grade}+\text{Extrinsic }*\text{ Group}+\text{Mindset }*\text{ Optimism}\\ +\text{Mindset }*\text{ Grade}+\text{Optimism }*\text{ Grade}\\ +\left(1|\text{DaysOff}\right)+\left(1|\text{School}\right) \end{array}$$

We then more precisely characterized the two groups, respectively. We thus tested which model better fit the data with the buildmer package from R for each group. This package performs backward stepwise elimination based on the change in log-likelihood (https://CRAN.R-project.org/package=buildmer). The likelihood ratio test is largely used to compare nested models and avoid overfitting (Glover and Dixon, 2004; Lewis et al., 2011).

The best model for group 1 followed equation 2:


(2) $$\begin{array}{l} \text{SqMATH }=\;1\;+\text{ Identified }+\text{ Grade }+\;(1\;|\text{ DaysOff})\\ +\;(1\;|\text{ School}) \end{array}$$

The best model for group 2 followed equation 3:


(3) $$\begin{array}{l} \text{SqMATH = Identified + Identified }*\text{ Extrinsic + Extrinsic +}\\ \text{Optimism + Optimism }*\text{ Mindset + Mindset + (1 | DaysOff)}\\ \text{+ (1 | School)} \end{array}$$

All descriptive statistics included the estimated coefficients (β), *t*-values (with approximate degrees of freedom following Satterthwaite), and 95% CI. *Post hoc* two-sampled, two-tailed *t*-tests were conducted on the average time spent on math homework. The threshold for statistical significance in all analyses was *p* < 0.05.

## Results

### Impact of School Closure vs. Schools Being Open During the COVID-19-Related Lockdown

**Figure 1:**
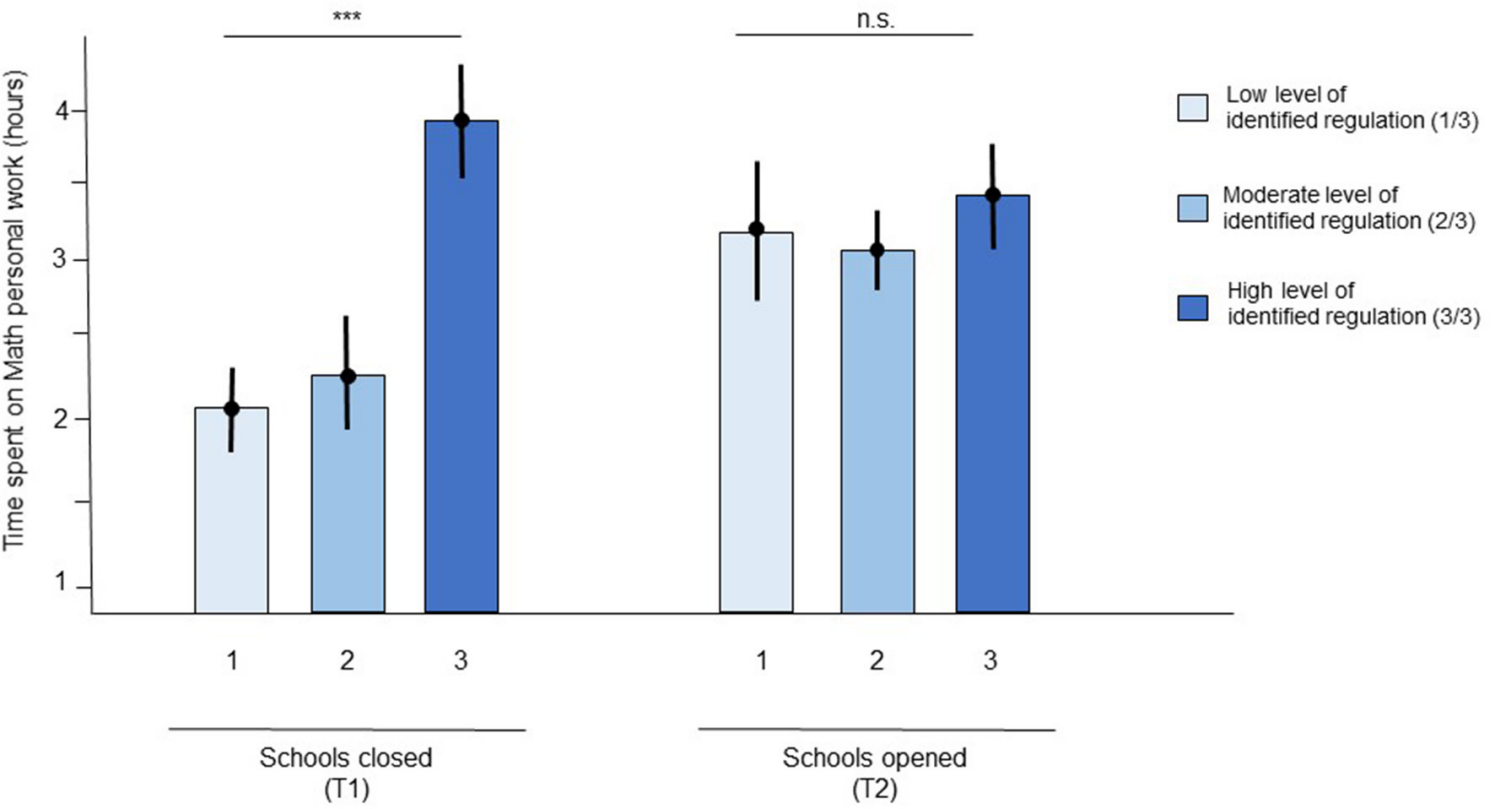
Time spent on personal work in Math was influenced by identified regulation only during the first lockdown with school closed. Means are represented by a dot and standard errors of the mean by the errors bars. *** indicates that the main effect of identified was significant with *p* < 0.01 during the lockdown with schools closed. n.s. indicates that the main effect of identified regulation was non-significant (*p* > 0.05) during the lockdown with schools opened.

A linear mixed-effects model of time spent on math homework showed a main effect of IR {β = 0.18, *t*_(141)_ = 2.31, *p* = 0.02, 95% CI [0.04, 0.32]}, and grade {β = −0.08, *t*_(141)_ = −2.04, *p* = 0.04, 95% CI [−0.16, −0.01]}, with non-significant effects of intrinsic regulation {β = 0.09, *t*_(141)_ = 1.27, *p* = 0.21, 95% CI [−0.04, 0.20]}, ER {β = −0.01, *t*_(141)_ = −0.21, *p* = 0.83, 95% CI [−0.12, 0.10]}, growth mindset {β = 2.1e-03, *t*_(141)_ = −2.04, *p* = 0.97, 95% CI [−0.11, −0.11]}, and optimism {β = −0.11, *t*_(141)_ = −1.79, *p* = 0.07, 95% CI [−0.23, 0.00]} (Table 2). Students who either had high levels of IR or were in earlier grades spent more time on math homework than students who had lower levels of IR or were in higher grades. Importantly, the main effect of IR was driven by a significant negative interaction between IR and group {β = −0.14, *t*_(141)_ = −2.02, *p* = 0.04, 95% CI [−0.27, −0.02]} (Figure 1). *Post hoc t*-tests showed that students with low IR worked less on math assignments and those with high IR worked more [*t*_(95)_ = 2.84, *p* = 3.30e-03] but only in the group tested during the COVID-19-related lockdown with schools closed. This difference was non-significant in the group tested during the COVID-19-related lockdown with schools open [*t*_(72)_ = 1.43, *p* = 0.08]. No other interactions were detected. Notably, no main effect of group was found {β = −0.03, *t*_(141)_ = −0.49, *p* = 0.63, 95% CI [−0.15, 0.09]}, suggesting that students spent a similar amount of time doing math homework when schools were closed and when they were open.

### School Engagement in Group 1, During a COVID-19-Related Lockdown With Schools Closed

We tested how time spent on math homework was determined during the first lockdown period with schools closed. The model that fits this dataset best included two regressors, namely, IR and grade (Table 3). Consistent with our main results, this model confirmed that IR predicted the engagement of students {β = 0.23, *t*_(94)_ = 2.89, *p* = 4e-3, 95% CI [0.07, 0.38]}. The more the students were driven by IR, the more they engaged in doing math homework. We also re-detected the main effect of grade {β = −0.12, *t*_(94)_ = −2.55, *p* = 0.01, 95% CI [−0.22, −0.03]}. *Post hoc t*-tests showed this main effect to be driven by significant differences between 6th and 8th graders [*t*_(47)_ = −2.96, *p* = 2.5e-3] and 6th and 9th graders [*t*_(42)_ = −2.73, *p* = 4.6e-3]. The differences between students in the 7th and 8th grades [*t*_(51)_ = −1.32, *p* = 0.09] and those in the 7th and 9th grades were borderline non-significant [*t*_(46)_ = −1.33, *p* = 0.09].

**Table 3:** Linear mixed effects model of time spent on math homework in *N* = 97 middle school students tested during the COVID-19 lockdown with schools closed.

**Fixed effects**	**β_i_**	**SE**	**DF**	**t**	***p***	**95%CI**
(Intercept)	1.78	0.07	94	24.33	2e-16	1.63–1.92
Identified	0.23	0.08	94	2.89	0.005	0.07–0.38
Grade	−0.12	0.05	94	−2.55	0.012	−0.22–0.03
**Random Effects**						
		Variance				
School (5 levels)	(*Intercept*)	0.00				
Days Off (4 levels)	(*Intercept*)	0.00				
Residual		0.52				
Observations = 97; REML = 219.1						

### School Engagement in Group 2, During a COVID-19-Related Lockdown With Schools Open

**Figure 2:**
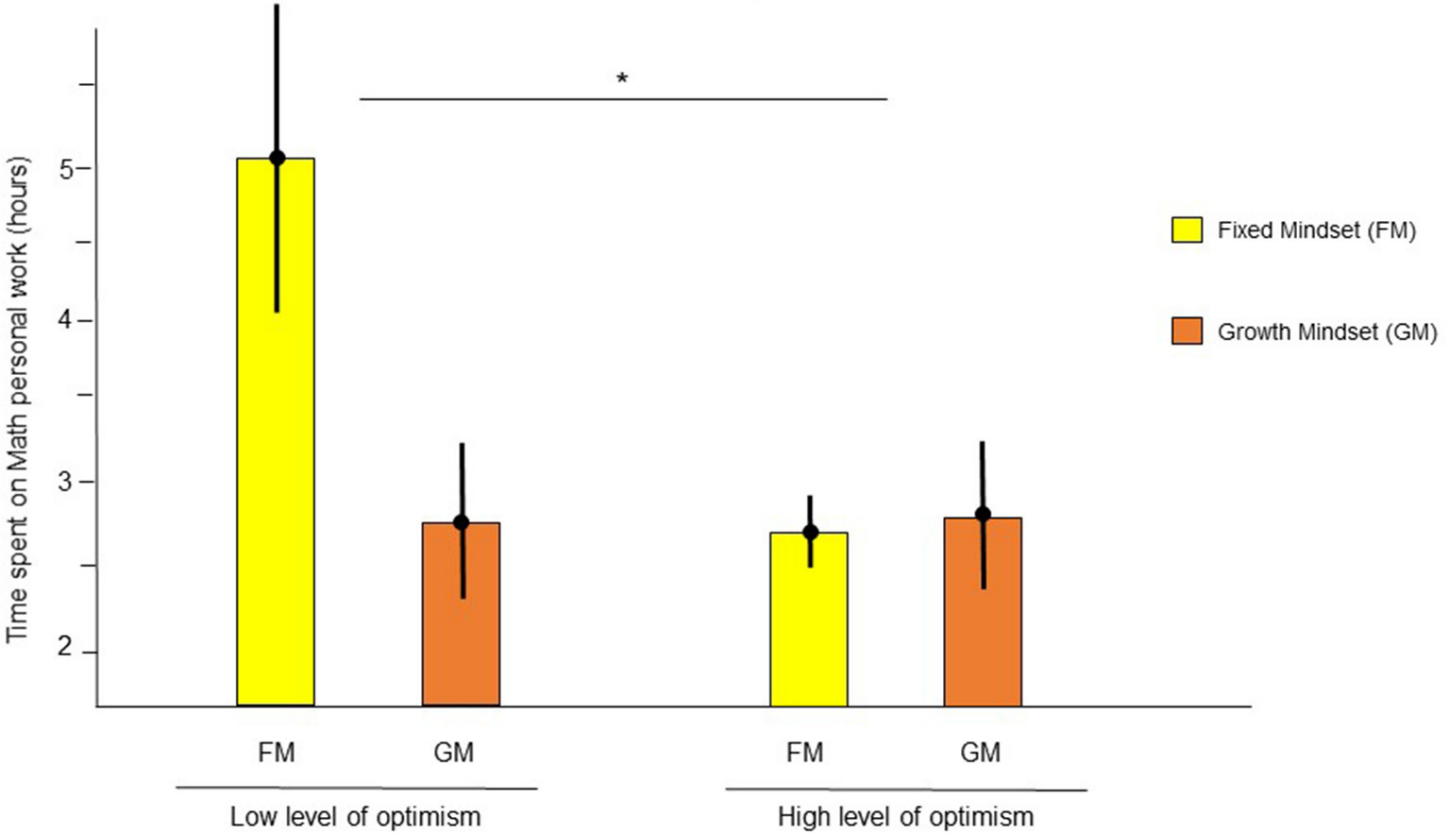
Time spent on personal work in Math was more impacted by mindset for pessimistic students than optimistic students. Means are represented by a dot and standard errors of the mean by the errors bars. *indicates that the interaction between mindset and optimism was significant with *p* < 0.05.

The statistical model that best described this dataset, which was collected during the second lockdown period with schools open, included four regressors, namely, identified and ER, optimism, and mindset, and two interaction regressors, namely, identified by ER and optimism by mindset (Table 4). There was a significant interaction between identified and ER (β = 0.15, *t*_(65)_ = 2.09, *p* = 0.04, 95% CI [7e-3, 0.29]}, which indicates that additional regulation by external gratification (reward, good scores) had a positive impact only for students with the highest level of IR [score of 3/3: *t*_(48)_ = 2.47, *p* = 0.01]. No significant interactions were found with ER on the time spent on math homework for students who scored medium or low on IR [score of 2/3: *t*_(15)_ = −2.10, *p* = 0.97; score of 1/3: *t*(4) = −1.07, *p* = 0.82]. Moreover, an interaction between optimism and mindset {β = 0.17, *t*_(65)_ = 2.05, *p* = 0.04, 95% CI [0.01, 0.34], Figure 2} indicated that students with a fixed mindset and low levels of optimism spent more time on math homework than students with low levels of optimism and a growth mindset and students with high levels of optimism and growth or a fixed mindset.

**Table 4:** Linear mixed effects model of time spent on math homework in *N* = 73 middle school students tested during the COVID-19 lockdown with schools open.

**Fixed effects**	**β_i_**	**SE**	**DF**	**t**	***p***	**95%CI**
(Intercept)	1.63	0.11	1.90	15.21	5e-03	1.38–1.86
Identified	0.10	0.08	64.5	1.29	0.20	−0.05–0.26
Extrinsic	0.12	0.08	64.7	1.49	0.14	−0.03–0.28
Optimism	−0.05	0.08	65.4	−0.70	0.49	−0.21–0.11
Mindset	−0.08	0.09	65.9	−0.96	0.34	−0.25–0.11
Identified * Extrinsic	0.15	0.07	65.0	2.09	0.04	0.007–0.3
Optimism * Mindset	0.17	0.08	64.6	2.05	0.04	0.01–0.34
**Random Effects**						
		Variance				
School (5 levels)	(*Intercept*)	0.01				
Days Off (4 levels)	(*Intercept*)	0.00				
Residuals		0.48				
Observations = 73 ; REML = 169.9						

## Discussion

This study explored the source of inter-individual differences in psychological states and how they affected school engagement in mathematics during the COVID-19 lockdown when schools were closed compared to when they were open. We explored the contribution of motivation regulation, intelligence mindset, and optimism and focused on the time students spent doing math assignments, as achieving good numeracy skills is one of the biggest challenges in middle school. Our results converged on the finding that the IR of motivation drove school engagement, specifically during the lockdown when schools were closed. This principal finding emphasizes the importance of school for developing competencies and constructing a professional path. Schools play a central role in the social construction and integration of adolescents (Vincent, 2008). Moreover, the academic and social aspects of school are not independent in middle school, as the social aspects of school have been shown to influence achievement and motivation (Day et al., 2014; Ladd and Kochenderfer-Ladd, 2016). Closing schools during the COVID-19 pandemic led to thousands of middle school students being deprived of appropriate study environments, as well as social interactions with peers and teachers. Crucially, our results show that such deprivation particularly affected students with a low level of identified motivation. Identified motivation implies that actions are driven (regulated) in coherence with his/her own attitudes, values, and needs. Thus, students who did not observe a value in mathematics in terms of their needs, attitudes, and values worked less during the first lockdown, when schools were closed than students surveyed during the lockdown of social and economic life, but with schools remaining open.

Overall, within the whole sample, younger students in the lower middle school grades spent more time on math homework than older students in the higher middle school grades, irrespective of whether the schools were closed or not. Such a decrease in engagement by grade is consistent with the well-documented decrease in performance and motivation in mathematics during middle school (Cayouette-Remblière and Moulin, 2019). Interestingly, the student group that was surveyed during the lockdown period with the schools open showed a positive impact on ER, specifically for students with high IR. This result is consistent with those of similar studies conducted in normal health and societal contexts that showed that ER is beneficial if coupled with greater personal motivation (Cameron et al., 2001; Cerasoli et al., 2014).

We did not find a positive main effect of growth mindset across all students, which is in contrast to the literature, which favors the hypothesis that a growth mindset positively affects school performance and engagement (Sarrasin et al., 2018). A recent impact study conducted on 23,000 French middle school students found that a growth mindset positively determined school performance in terms of the grades of the final examination but not engagement in terms of time spent on homework (Huillery et al., 2021). Our findings are consistent with this conclusion. However, more studies are needed to better determine the mechanisms through which mindset affects school engagement. Future studies should consider asking students to rate what they think about their intelligence in a specific field, such as math, to test the hypothesis of stronger mindset effects on school engagement in a specific field.

We did not find a main effect of optimism, which runs contrary to previous work showing a positive effect of optimism on school achievement counteracting dropping out (Solberg Nes et al., 2009). These findings suggest that potential variables that mediate the effects of optimism on school achievement do not involve school engagement. Interestingly, although there were no overall main effects of growth mindset or optimism, the two variables interacted when predicting school engagement for the students who were surveyed during the second lockdown, with schools open. Students with a fixed mindset together with more pessimistic views of the future worked more on math exercises than optimistic students with a fixed mindset. There was no difference in school engagement between pessimistic and optimistic students who adopted a growth mindset. For fixed mindset students with low optimism scores, and thus more pessimistic views of the future, focusing on work can be a coping strategy to deal with emotions emerging from a context with high uncertainty concerning the future (emotion-focused coping, Lazarus and Folkman, 1984). However, the previous study has also shown that the study environment moderates the impact of mindset in school engagement. We showed that adopting a growth mindset is not sufficient to change attitudes and engagement toward scholarship if the study environment does not value and encourage effort and training (Walton and Yeager, 2020). The idea that innate abilities in science are necessary to be good in math is widely shared (Gunderson et al., 2017). It is therefore possible that leaving schools open during an adverse lifetime event, such as the COVID-19-related lockdown, created a study environment that allowed fixed mindset students to work harder to counteract worries about the potential negative effects of the pandemic on their future life and career. There is a small but growing body of research that has studied the role of coping strategies during the COVID-19 pandemic, but it has focused mainly on dealing with the increased health risks of COVID-19 (Baloran, 2020; Gerhold, 2020). Future studies are needed to understand the role of going to school during adverse life events and the complex interactions between anxiety and fears and growth vs. fixed mindsets and optimism.

Although our results highlight the importance of IR, in particular, when schools are closed, our study also involves some limitations such as the sample size and sample selection. In fact, student participation was voluntary. Thus, we cannot exclude that the responding participants were also those who were the most motivated. In addition, although the pandemic situation was similar during the two lockdown periods, the first lockdown was a novel, unprecedented experience, whereas it was more familiar when students experienced it the second time. The COVID-19 pandemic-related lockdowns of social and economic life provided a unique opportunity to study the effects of this collective adverse real-life event and how its potential negative effects on school engagement were further enhanced by school closure or attenuated by keeping schools open. Obtaining such data, for the first time, comes with the absence of randomization and the resulting sequential testing biases. We could at least partially rule out confounders due to such biases on school engagement *per se* because school engagement was matched between the two groups. However, we called for more studies to explore the impact of familiarity with experiencing a stressful adverse life-event situation and that of sequential surveying on the interactions between school engagement and motivation regulation, mindset, and optimism.

Previous studies have shown a positive impact of identified motivation on school performance and for avoiding dropout. Importantly, this positive effect was observed across grades and education levels from elementary school (Burton et al., 2006) and high school (Nishimura et al., 2011) to university (Black and Deci, 2000). It also generalizes across diverse educational subjects such as science (Black and Deci, 2000), physical (Boiché et al., 2008), and language education (Joe et al., 2017). Thus, it is very likely that identified motivation has played an important role in school engagement for students from different schools and grades, especially during such an adverse event as the combination of lockdown and school closure. It is possible that during the lockdown with schools closed, time spent on homework was also influenced by diverse other, more pragmatic factors such as access to a computer, food, and electricity, adult supervision, or attending online courses with teachers. These personal data, however, could not be collected due to reglementary policies assuring the full anonymity of the survey responses. However, during the first lockdown, we had the opportunity to collect some data about COVID-19 fear, frustration linked to experiencing a lockdown, and the number of people living under the same roof during the lockdown. A correlational analysis reported in Supplementary Material revealed that the number of people under the same roof had a significant, positive impact on the time spent on mathematics homework. Further studies including more demographic data and additional data from teachers and students with no access to the Internet are thus important to better understand how strong the impact of identified motivation is when students are deprived of direct interactions with teachers and classmates.

In this study, we focused on the time students spent on mathematics homework as an indicator for school engagement in mathematics following the participation-identification model of school engagement (Finn, 1989; Finn and Zimmer, 2012). The model proposes that school engagement of students can be decomposed into a behavioral component, i.e., participation, and a psychological component, i.e., identification, both components reinforcing each other (Finn, 1993; Virtanen et al., 2021). However, participation is more strongly linked to school dropout (Archambault et al., 2009), which is more likely to occur during adverse life events (Shahar et al., 2009). Given these interactions and the adverse context of the COVID-19 pandemic, we considered that the behavioral participation component of school engagement was most relevant. We further reason that participation can be approximated by how much time the students allotted for mathematics homework, which reflects a specific type of school engagement, motivated by the commonly shared concern of improving math skills throughout middle schools. However, our findings in this specific type of school engagement do not allow inferences on how identified motivation determines school engagement more generally. More work is, therefore, needed to identify the general behavioral components of school engagement under adverse life events, when students are deprived of direct interaction with peers and teachers at school. Moreover, it was not possible to take into account how much attendance to online courses determined the time spent on math homework. When schools were closed during the lockdown, there were no guidelines concerning online courses, and the conditions to attend these courses varied much across teachers, schools, and households. We also cannot rule out confounds by the sources of inter-individual differences in school performance and time management. The previous study has shown that spending more time doing homework does not always lead to better school performances (De Jong et al., 2010; Kitsantas et al., 2011), and students with lower math skills may spend more time than their peers for the same amount of homework. The scope of our findings is limited to the behavioral participation component of school engagement in mathematics. We, therefore, encouraged more studies to better understand how school performance and school engagement interact and are moderated by inter-individual differences in the capacity to manage time.

## Conclusion

Our findings shed light on the psychological determinants of school engagement when schools are closed during a pandemic. In particular, we showed that the IR of motivation predicted school engagement in mathematics. This finding provides new evidence that may be useful for the implementation of wise interventions (Walton and Wilson, 2018), such as utility value interventions that help students to understand how valuable knowledge can be for them outside the classroom (Hulleman et al., 2010, 2017; Harackiewicz et al., 2016; Canning et al., 2018). These interventions could easily be carried out online as they usually ask students to write a short essay about the relevance of a specific knowledge they learn at school. Results show that even such a short exercise could help students to change their perception of school subjects and help them to increase interest and grades (Hulleman et al., 2010; Harackiewicz et al., 2016; Canning et al., 2018). Targeting identified motivation with such wise interventions during adverse lifetime events, such as a pandemic-related lockdown and school closure, could therefore be of relevance to maintain the engagement of students in studying for school during such challenging periods.

## Data Availability Statement

The datasets presented in this study can be found in online repositories. The names of the repository/ repositories and accession number(s) can be found below: https://github.com/leacombette/COMBETTECAMENENROTGESCHMIDT.

## Ethics Statement

Ethical review and approval was not required for the study on human participants in accordance with the local legislation and institutional requirements. Written informed consent to participate in this study was provided by the participants' legal guardian/next of kin.

## Author Contributions

LC, J-YR, and LS conceived and designed the study. LC collected and analyzed the data. EC assisted with data analyses. LS and J-YR supervised the data analysis. LC and LS wrote the first draft of the manuscript. All authors contributed to the final manuscript.

## Funding

LC was supported by the ANRT and Energie Jeunes Foundation.

## Conflict of Interest

The authors declare that the research was conducted in the absence of any commercial or financial relationships that could be construed as a potential conflict of interest.

## Publisher's Note

All claims expressed in this article are solely those of the authors and do not necessarily represent those of their affiliated organizations, or those of the publisher, the editors and the reviewers. Any product that may be evaluated in this article, or claim that may be made by its manufacturer, is not guaranteed or endorsed by the publisher.
