# Supplementary material for: Identified Motivation as a Key Factor for School Engagement During the COVID-19 Pandemic-Related School Closure
Source: Front Psychol. 2021 Nov 16;12:752650. doi: 10.3389/fpsyg.2021.752650 (PMC8635061; doi:10.3389/fpsyg.2021.752650)
Supplement: Supplementary file 1 [file Data_Sheet_1.docx]

Supplementary Material

#

# Supplementary analysis

# Cluster analysis of motivation regulation and effects of motivational profiles on school engagement

1.2. Analysis of contextual factors

1. Supplementary data: Supplementary tables 1 – 3
2. Supplementary material: Questionnaires
   1. Elementary School Motivation Scale
   2. Revised Life Orientation Test
   3. Growth Mindset Scale

4. Supplementary references

**1. Supplementary analysis**

# 1.1 Cluster analysis of motivation regulation and effects of motivational profiles on school

# engagement

In our main analysis, we explored the impact of intrinsic, identified, and extrinsic regulation of motivation on school engagement. Nevertheless, instead of testing the relative weight of each of these types of regulation, another typical approach is to test the impact of motivational profiles, that is, the impact of specific combinations of intrinsic, identified, and extrinsic regulation (Boiché, Sarrazin, Grouzet, Pelletier & Chanal, 2008; Vansteenkiste, Sierens, Soenens, Luyckx & Lens, 2009; Howard, Gagné, Morin, & Van den Broeck, 2016; Gillet, Morin & Reeve, 2017). Following the same logic as that used for our main analysis, we first analyzed motivational profiles for the overall sample (group 1 tested during a lockdown with schools closed, T1 + group 2 tested during a lockdown with schools kept open, T2) before to more precisely search for the motivational profiles that characterized students in T1 and T2. Motivational profiles were created following two steps. First, the optimal number of clusters was determined by the Gap-statistic (Tibshirani, Walther & Hastie, 2001) using the clusGap function from the cluster package in R. Then, we used the unweighted pair group method with the arithmetic mean (UPGMA) hierarchical clustering method to create clusters (Sokal & Michener, 1958).

Analyses of the overall sample highlighted the existence of two distinct motivational profiles (Table SI1) that different in the levels of identified and intrinsic regulation. While extrinsic regulation was at the same level, the students within the first cluster displayed, higher intrinsic (t(45) = 8.31, p = 1e-10) and higher identified regulation (t(30) = 11.96, p = 5e-13) compared to the students in cluster 2. One-tailed t-test analyses further revealed that these students spent more time working on math (t(42)=2.48, p= 0.02).

**Students surveyed during the lockdown with schools closed (T1).**

Analyses for the T1 sample highlighted the existence of eight distinct motivational profiles (Table SI2). Analysis of variance (ANOVA) showed that these eight motivational profiles affected the time spent on mathematics (F(1) = 5.52, p = 0.02). More precisely, students with no intrinsic regulation (score equal to zero), low identified regulation, and high extrinsic regulation (cluster 4: ẞ = -0.9, t(89) = -2.18 p = 0.03) and students with low intrinsic regulation, low identified regulation, and low extrinsic regulation (cluster 7: ẞ = -0.72 , t(89) = -1.57, p = 7e-3) performed significantly worse than students displaying other motivational profiles.

**Students surveyed during the lockdown with schools kept open (T2).**

Analyses for the T2 sample highlighted the existence of five distinct motivational profiles (Table SI3). An ANOVA showed that in this sample the five motivational profiles did not affect time spent working on mathematics when schools remained open (F(1) = 3.02, p = 0.09).

# Analysis of contextual factors

# In the group of students surveyed schools were closed, data about three contextual factors was collected: (1) fear of COVID-19, (2) frustration related to the lockdown, and (3) number of persons living under the same roof during the lockdown. These data could not be collected in the second group (T2, with schools open), because of logistical issues. Indeed, school principals, teachers and students were under a lot of pressure during the lock down with schools open, because of the sanitary restrictions. We therefore shortened the survey removing some of the socio-demographic questions. The association between these three measures and time spent on math homework in the sample surveyed when schools were closed was analyzed using PEARSON’s correlation with the cor.test function of the stat package in R. No significant correlation was found between time spent on mathematic homework and the fear of COVID-19 (r = 0.06, p = 0.87), or the frustration of being locked down (r = 0.14, p = 0.14). Interestingly, a significant positive correlation indicated that students in households with more family members spent more time on homework (n = 97, r =0.25, p = 0.01). This result may appear surprising because one could argue that students with larger families are more often requested to look after sibling and thus have less time for personal schoolwork. However, our finding is somewhat in line with previous research in sociology, that has shown that family size could influence positively students’ school performance. For example, students with immigrant background have been shown to engage more by transmitting their novel knowledge acquired in the French school system to younger siblings, which is beneficial for the overall school performance (Ichou, 2016). However, we do not have demographic data about the immigrant/non-immigrant status of families. Moreover, our question (How many family members are you locked down with?) does not allow us to know whether, for example, a family of 6 people is composed of two parents and four kids or two parents, two grandparents and two kids. Further studies should explore more precisely the impact of family composition on time spent on homework.

**2. Supplementary data**

**2.1 Supplementary table 1:**

| **Table SI1.** Average level of intrinsic, identified, and extrinsic regulation for each cluster in overall sample of N=170 participants. | | | |
| --- | --- | --- | --- |
|  | **Cluster 1**  n=144 | **Cluster 2**  n = 26 | Two sampled, one tailed t-test |
| **Intrinsic regulation** | m = 1.8, sem = 9e-2 | m = 0.4, sem = 0.1 | t(45) = 8.3, p = 1e-10 |
| **Identified regulation** | m = 2.8, sd = 3e-2 | m = 1.6, sem = 0.1 | t(30) = 11.9, p = 5e-13 |
| **Extrinsic regulation** | m = 1.0, sd = 8e-2 | m = 1.1, sem = 0.2 | t(31) = -0.2, p = 0.8 |

**2.2 Supplementary table 2:**

| **Table SI2.** Average level of intrinsic, identified, and extrinsic regulation for each cluster in the student sample (N=97) surveyed during the COVID-19 related lockdown with schools closed (T1 sample). | | | | | | | | |
| --- | --- | --- | --- | --- | --- | --- | --- | --- |
|  | **Cluster 1**  n = 19 | **Cluster 2**  n = 30 | **Cluster 3**  n = 13 | **Cluster 4**  n = 3 | **Cluster 5**  n = 8 | **Cluster 6**  n = 16 | **Cluster 7**  n = 8 | **Cluster 8**  n = 3 |
| **Intrinsic regulation** | m=2.37, sem= 0.1 | m=2.27, sem= 0.1 | m=0.92, sem=0.2 | m=0.00, sem=0.0 | m=2.5, sem=0.2 | m=0.12, sem=0.1 | m=0.6, sem=0.4 | m=0.33, sem=0.3 |
| **Identified regulation** | m=3.00, sem= 0.0 | m=3.00 , sem=0.0 | m=2.00, sem=0.0 | m=2.00, sem=0.0 | m=2.00, sem=0.0 | m=3.00, sem=0.0 | m=1.00, sem=0.0 | m=3.00, sem=0.0 |
| **Extrinsic regulation** | m=2.32, sem=0.1 | m=0.04, sd= 0.1 | m=1.00, sem= 0.1 | m=2.33, sem= 0.3 | m=0.25, sem= 0.2 | m= 0.56, sem= 0.1 | m=0.20, sem=0.2 | m=2.00, sem=0.0 |

Considering students to have a low level of regulation if their level is below the group median and high if it is above, the clusters can be described as follows:

Cluster 1: High intrinsic regulation - High identified regulation - High extrinsic regulation

Cluster 2: High intrinsic regulation - High identified regulation - Low extrinsic regulation

Cluster 3: Low intrinsic regulation - Low identified regulation - High extrinsic regulation

Cluster 4: No intrinsic regulation - Low identified regulation - High extrinsic regulation

Cluster 5: High intrinsic regulation - Low identified regulation - Low extrinsic regulation

Cluster 6: Low intrinsic regulation - High identified regulation - Low extrinsic regulation

Cluster 7: Low intrinsic regulation - Low identified regulation - Low extrinsic regulation

Cluster 8: Low intrinsic regulation - High identified regulation - High extrinsic regulation

**2.3 Supplementary table 3:**

| **Table SI3.** Average level of intrinsic, identified, and extrinsic regulation for each cluster in the students surveyed during the lockdown with schools kept open (N=73, T2 sample). | | | | | |
| --- | --- | --- | --- | --- | --- |
|  | **Cluster 1**  n = 30 | **Cluster 2**  n = 5 | **Cluster 3**  n = 16 | **Cluster 4**  n = 7 | **Cluster 5**  n = 15 |
| **Intrinsic regulation** | m=2.60, sem=0.1 | m=0.00, sem=0.0 | m=1.25, sem=0.2 | m=2.43, sem=0.2 | m=0.33, sem=0.1 |
| **Identified regulation** | m=3.00, sem=0.0 | m=1.00, sem=0.0 | m=2.62, sem=0.1 | m=1.86, sem=0.1 | m=2.67, sem=0.1 |
| **Extrinsic regulation** | m=0.93, sem=0.1 | m=1.40, sem=0.5 | m=2.37, sem=0.1 | m= 0.28,  sem= 0.2 | m=0.47,  sem= 0.1 |

Considering students to have a low level of regulation if their level is below the group median and high if it is above, the clusters can be described as follows:

Cluster 1: High intrinsic regulation - High identified regulation - Low extrinsic regulation

Cluster 2: No intrinsic regulation - Low identified regulation - High extrinsic regulation

Cluster 3: Low intrinsic regulation - Low identified regulation - High extrinsic regulation

Cluster 4: High intrinsic regulation - Low identified regulation - Low extrinsic regulation

Cluster 5: Low intrinsic regulation - Low identified regulation - Low extrinsic regulation

**3. Supplementary material: Questionnaires**

**3.1 Elementary School Motivation Scale**

I like Maths

- Yes
- No

Maths interests me a lot

- Yes
- No

I do maths even when I don’t have to

- Yes
- No

I can learn many useful things by doing maths

- Yes
- No

I choose to do maths to learn many things

- Yes
- No

In life, it’s important to learn how to do maths

- Yes
- No

I do maths to get a nice reward

- Yes
- No

I do maths to please my parents or my teacher

- Yes
- No

I do maths to show others how good I am

- Yes
- No

**3.2 Revised Life Orientation Test**

In uncertain times, I usually expect the best.

- Totally disagree
- Disagree
- Agree
- Totally Agree

It’s easy for me to relax.

- Totally disagree
- Disagree
- Agree
- Totally Agree

If something can go wrong for me, it will.

- Totally disagree
- Disagree
- Agree
- Totally Agree

I’m always optimistic about my future.

- Totally disagree
- Disagree
- Agree
- Totally Agree

I enjoy my friends a lot.

- Totally disagree
- Disagree
- Agree
- Totally Agree

It’s important for me to keep busy.

- Totally disagree
- Disagree
- Agree
- Totally Agree

I hardly ever expect things to go my way.

- Totally disagree
- Disagree
- Agree
- Totally Agree

I don’t get upset too easily.

- Totally disagree
- Disagree
- Agree
- Totally Agree

I rarely count on good things happening to me.

- Totally disagree
- Disagree
- Agree
- Totally Agree

Overall, I expect more good things to happen to me than bad.

- Totally disagree
- Disagree
- Agree
- Totally Agree

**3.3 Growth Mindset Scale**

You have a certain amount of intelligence, and you can’t really do much to change it.

- Totally agree
- Agree
- Agree a little
- Disagree a little
- Disagree
- Totally disagree

Your intelligence is something about you that you can’t change very much.

- Totally agree
- Agree
- Agree a little
- Disagree a little
- Disagree
- Totally disagree

You can learn new things, but you can’t really change your basic intelligence.

- Totally agree
- Agree
- Agree a little
- Disagree a little
- Disagree
- Totally disagree

**4. Supplementary references:**

Boiché, J., Sarrazin, P. G., Grouzet, F. M., Pelletier, L. G., & Chanal, J. P. (2008). Students' motivational profiles and achievement outcomes in physical education: A self-determination perspective. Journal of educational psychology, 100(3), 688.

Gillet, N., Morin, A. J., & Reeve, J. (2017). Stability, change, and implications of students’ motivation profiles: A latent transition analysis. Contemporary Educational Psychology, 51, 222-239.

Howard, J., Gagné, M., Morin, A. J., & Van den Broeck, A. (2016). Motivation profiles at work: A self-determination theory approach. Journal of Vocational Behavior, 95, 74-89.

Ichou, M. (2016). Diluting or Transmitting Resources?.

Tibshirani, R., Walther, G., & Hastie, T. (2001). Estimating the number of clusters in a data set via the gap statistic. Journal of the Royal Statistical Society: Series B (Statistical Methodology), 63(2), 411-423.

Vansteenkiste, M., Sierens, E., Soenens, B., Luyckx, K., & Lens, W. (2009). Motivational profiles from a self-determination perspective: The quality of motivation matters. Journal of educational psychology, 101(3), 671.

**
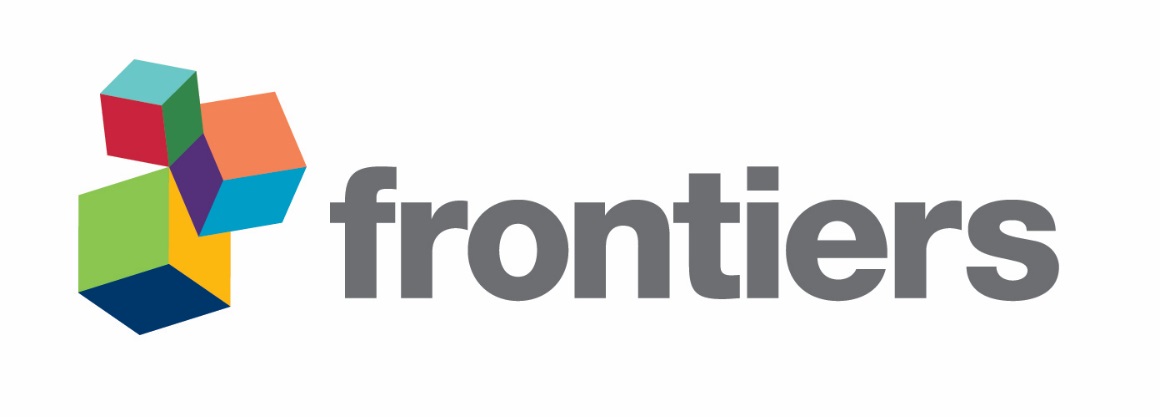
**
